# Effect of a WeChat-Based Hybrid Intervention on the Adaptation Outcomes of People Living With HIV/AIDS: Pilot Randomized Controlled Trial

**DOI:** 10.2196/65268

**Published:** 2025-04-03

**Authors:** Honghong Wang, Ziqi Qin, Yixuan Li, Yuqiong Duan, Qiaoyue Lu, Xueling Xiao

**Affiliations:** 1 Xiangya School of Nursing Central South University Changsha China

**Keywords:** HIV/AIDS, quality of life, acceptance of illness, mental health, randomized controlled trial, mobile phone

## Abstract

**Background:**

People living with HIV/AIDS face multiple challenges that collectively impede their adaptation outcomes. These outcomes include quality of life (QoL), acceptance of illness, mental health (including symptoms of anxiety and depression), and antiretroviral therapy (ART) adherence. While existing evidence addresses specific challenges, it often overlooks the interactions among the various problems people living with HIV/AIDS encounter. The comprehensive-task disease management framework and positive self-management framework provide a theoretical basis for understanding the adaptation process. A culturally tailored, theory-based intervention may be necessary and effective in facilitating better adaptation outcomes for people living with HIV/AIDS.

**Objective:**

This study aimed to evaluate the effect of a hybrid intervention called AiCare (Adaptation intervention with Comprehensive-task disease management framework to achieve renormal life) on improving QoL, acceptance of illness, mental health (anxiety and depression), and ART adherence among people living with HIV/AIDS in China.

**Methods:**

We conducted a 2-arm randomized controlled trial, recruiting 92 people living with HIV/AIDS from an HIV clinic in Hunan, China. Participants were randomly assigned in a 1:1 ratio to either the control group (receiving standard care) or the intervention group (receiving AiCare in addition to standard care). All analyses were performed from an intention-to-treat perspective. Sociodemographic and HIV-specific clinical characteristics, along with key adaptation outcomes—including QoL, acceptance of illness, mental health (anxiety and depression), and ART adherence—were assessed at baseline (T0), post intervention (T1), and 3 months post intervention (T2). We used generalized estimating equation models and difference-in-difference analysis to evaluate the interventions’ effects.

**Results:**

The difference-in-difference model showed that at T1, the intervention group experienced significant improvements compared to the control group. QoL increased by 6.35 (95% CI 2.62-10.93, *P*=.001), acceptance of illness improved by 4.49 (95% CI 2.29-6.68, *P*<.001), and anxiety decreased by 2.15 (95% CI 1.19-3.11; *P*=.01). At T2, the intervention group’s improvement in QoL was not statistically significant (β 3.62, 95% CI –1.53 to 8.77; *P*=.17). However, acceptance of illness remained significantly improved by 3.65 (95% CI 1.22-6.08; *P*=.003), and anxiety decreased by 1.58 (95% CI 0.42-2.74; *P*=.007). No significant changes were observed in depression or ART adherence between the intervention and control groups. Feedback regarding the AiCare program indicated its acceptability and feasibility.

**Conclusions:**

The AiCare program demonstrated promising effects in improving disease adaptation outcomes among people living with HIV/AIDS, notably in enhancing QoL, fostering acceptance of illness, and mitigating anxiety symptoms. These findings underscore the hybrid program’s potential clinical utility to facilitate the adaptation of people living with HIV/AIDS.

**Trial Registration:**

Chinese Clinical Trial Registry ChiCTR2400087255; https://www.chictr.org.cn/showproj.html?proj=220729

## Introduction

People living with HIV/AIDS have experienced a significant increase in survival rates, as antiretroviral therapy (ART) has transformed HIV from a fatal disease into a manageable chronic condition [1,2]. In 2023, there were a total of 39.9 million people living with HIV. Meanwhile, the number of new infections continues to rise [3]. According to the Chinese Center for Disease Control and Prevention, as of June 30, 2024, there were 1,329,127 people living with HIV/AIDS in China [4]. These people living with HIV/AIDS are provided with free ART, which consists exclusively of publicly funded, domestically produced medications [5,6]. As a result, most people living with HIV/AIDS rely on free options, such as efavirenz, or lopinavir, or ritonavir, both of which are associated with higher risks of adverse effects [7,8]. Additionally, people living with HIV/AIDS face significant social discrimination and internalized stigma, which are compounded by conservative cultural values, negatively impacting their mental health and treatment adherence [9]. Moreover, people living with HIV/AIDS experience the accumulation of comorbid conditions, including cardiovascular disease and obesity [10,11], and psychological distress such as anxiety [12] and depression [13]. They also face the burden of social and internalized stigma [14], vocational disparities [15], and other challenges, all of which further hinder their ability to adapt to living with HIV.

According to the comprehensive task-based model [16], the adaptation outcomes of people living with HIV/AIDS refer to their ability to accept HIV as part of their lives and achieve a new state of social and mental balance, thereby attaining a relatively satisfactory life status. This definition underscores the multidimensional nature of adaptation outcomes, encompassing quality of life (QoL), acceptance of illness, mental health, and ART adherence [17-19]. Besides, prior studies have shown that acceptance of illness [20] and mental health status [21-23], particularly levels of depression and anxiety, are crucial factors influencing the overall QoL for people living with HIV/AIDS; ART adherence plays a critical role in physical QoL [24]. By combining these variables—QoL as the central adaptation outcome, with acceptance of illness, anxiety, and depression, and ART adherence as indicators—this study provides a theoretically grounded and empirically supported holistic view of adaptation of people living with HIV/AIDS.

Mobile health (mHealth) provides an optimal foundation for delivering care to people living with HIV/AIDS at a lower cost compared to purely offline interventions [25-27]. However, an extensive synthesis of systematic reviews has highlighted a notable gap in the literature [28,29]: the majority of existing intervention studies have not specifically aimed to address QoL and have shown the equivocal nature of the effect of interventions on QoL. Only four of the ten studies showed improved QoL, while the others showed neutral or even deteriorated outcomes. Furthermore, interventions tailored to improve the QoL of people living with HIV/AIDS within the cultural context of China are scarce [30,31]. Most existing intervention studies focus on one specific aspect of adaptation outcomes among people living with HIV/AIDS, such as ART adherence [18,19,32] or mental health [21-23]. Although evidence shows a strong relationship between acceptance of illness and QoL among people living with HIV/AIDS [20], few studies have adopted acceptance of illness as an adaptation outcome measure. Given the controversial outcomes and the lack of China’s culturally tailored studies, it is imperative to develop further intervention strategies that specifically target the holistic view of adaptation outcomes.

Furthermore, the limitations of purely web-based interventions limit their effectiveness in fostering adaptation outcomes among people living with HIV/AIDS [33]. The inaccessibility of mHealth and the digital divide may contribute to lower participation and higher dropout rates [34]. Some studies have indicated that the lack of face-to-face care is another disadvantage, as individual-to-individual care cannot fully replace the need for personal contact [35,36]. Additionally, patients often expect immediate responses in mHealth interventions, which may limit the efficacy of these interventions and how participants perceive them [37].

Therefore, to address the lack of adaptation-outcome–tailored intervention among people living with HIV/AIDS in China and explore a feasible way to enhance the efficacy of mHealth, we developed this AiCare (Adaptation intervention with Comprehensive-task disease management framework to achieve renormal life) program based on literature review, theories, expert consultation, and preliminary studies [16-20]. In this study, we integrated offline face-to-face modules with mHealth to address limitations like lack of personal contact and digital barriers [34,36,37]. This randomized controlled trial was to test the feasibility and effect of the AiCare program on the adaptation outcomes among people living with HIV/AIDS. This study offers empirical support for a hybrid intervention approach to enhance the adaptation of people living with HIV/AIDS in a context similar to that of China.

## Methods

### Study Design

This study used a 2-arm randomized controlled trial, conducted in Changsha, China, from June to December 2020. Eligible participants were randomly allocated in a 1:1 ratio to either the hybrid intervention group, which received the AiCare program in addition to standard care, or the control group, which received standard care alone (see the CONSORT [Consolidated Standards of Reporting Trials] flowchart in the *Results* section and CONSORT checklist in Multimedia Appendix 1).

### Setting and Participants

We enrolled participants consecutively from the HIV clinic of the First Hospital of Changsha, recognized as the principal treatment facility for HIV in the capital city of Hunan province. Serving a diverse population, this clinic offers comprehensive medical services to more than 8000 people living with HIV/AIDS from municipalities throughout the province. Eligibility criteria for participation included (1) confirmed HIV infection; (2) initiation of ART; (3) age of 18 years or older; (4) the presence of negative adaptation outcomes, defined by specific cutoff scores on validated scales—namely, a score of ≥3 on the 4-item Patient Health Questionnaire (PHQ-4) for anxiety and depression, a score of ≤30 on the Acceptance of Illness Scale (AIS), or a score of ≤10 on the Center for Adherence Support Evaluation (CASE) Index; and (5) using WeChat. We excluded those who (1) were unable to communicate effectively, (2) lacked access to a smartphone, (3) had physical disabilities that impeded participation, (4) were pregnant, or (5) were engaged in other interventional studies, and (6) were unable to read or listen to the materials sent via WeChat (ie, short articles, audios, and posters).

Two trained research assistants (YL and ZQ) invited the potentially eligible participants in the waiting area of the HIV clinic to complete initial screening interviews and a screening questionnaire to determine whether they met the inclusion and exclusion criteria described above. They provided the eligible participants with a document about the AiCare program and further explained the details to those who were willing to join. After providing written informed consent, the participants finished the baseline survey on a web-based survey platform with the assistance of the research assistants [38]. The recruitment period was from June to July 2020, and the intervention experiment lasted for 10 weeks after the completion of the recruitment. Data were collected at baseline (T0), post intervention (T1), and 3 months post intervention (T2).

We adopted PASS 18.0 (NCSS, LLC) to compute the sample size. We assumed α=.05, β=.80, and an anticipated increase of 10 points of QoL; the SD for the people living with HIV/AIDS without intervention was 11.36 based on our preliminary survey [39], and the SD for PWLH receiving intervention was hypothesized as the same as that of the general population (17.46) [40]. The calculated sample size was 36 for each group, and allowing for 20%-30% attrition, the final sample size was 45-52.

### Randomization and Masking

Post baseline assessment, participants were randomly allocated to either the intervention group or the control group using a randomization list generated by SPSS (version 25.0; IBM Corp), with a seed value set at 202007. The randomization process was stratified by gender. Owing to the intrinsic nature of the intervention, blinding of participants and the interventionist was not viable. Nevertheless, the data collection team and the statistical analyst were blinded to the group allocation to mitigate potential bias.

### Procedure

#### Overview

Following allocation, all participants were instructed to follow the interventionist’s individual and official WeChat account, a social media platform with extensive prevalence and use in China.

#### Control Group

Participants in the control group received standard care. A clinic nurse offered free counseling to people living with HIV/AIDS during their clinic visits for physical examinations and ART medications. Additionally, a toll-free landline counseling service was made available by the same nurse, operational from Monday to Saturday during working hours (8:30 AM to 11:50 AM and 2 PM to 5 PM). Participants were also granted access to free counseling via WeChat, facilitated by a research team member (XX).

#### Intervention Group

As shown in Textbox 1, the hybrid intervention group received both the AiCare program and standard care, which spanned 10 weeks. The program comprised 3 individual sessions and 8 web-based sessions conducted via WeChat.

**The contents of the AiCare (Adaptation intervention with Comprehensive-task disease management framework to achieve renormal life) program.**

**Week 1 (offline)**

- Establishing trust and understanding disease adaptation
  Project introduction
  Knowledge of HIV infection, including disease progression and life expectancy
  Digital discussions based on the baseline questionnaire to determine personalized patient needs
  Introduction to and guidance on relaxation techniques
  Guidance on the use of the self-management checklist
  Importance of ART (antiretroviral therapy) medication adherence (consider simplifying if appropriate)

**Week 2 (web-based)**

- Self-management checklist
  Review of how to use the self-management checklist
- Importance and precautions of ART adherence
  Techniques to prevent others from knowing about the medication
  How to handle missed doses, makeup doses, and adjust medication times
  How to achieve undetectable=untransmittable status through effective ART
- Relaxation techniques
  Guided imagery relaxation with grasslands, lakes, and mountains

**Week 3 (web-based)**

- Management of common symptoms
  Basic principles of symptom management
  How to observe and manage common symptoms
- Importance of regular check-ups
  Importance and interpretation of CD4+ lymphocyte count and viral load testing
  Guide people living with HIV/AIDS to decide the frequency of the above tests based on their economic situation
  Importance and frequency of other tests such as liver and kidney function tests
- Relaxation techniques
  Breathing exercises and visualization training

**Week 4 (web-based)**

- Healthy lifestyle
  Principles and precautions of diet and exercise
  Impact of drugs, alcohol, and tobacco
  Management of sexual life
  Techniques for dealing with common problems in daily life
- Learning to accept the disease
  How to view disease
  Understanding that disease, like anything else, is uncertain
  Small strategies for accepting disease
- Relaxation techniques
  Body scan relaxation technique

**Week 5 (offline, or voice or video calls**

- Midterm feedback
  Understanding the problems that people living with HIV/AIDS have faced in the past month
  Summary of previous interventions and relaxation techniques
  Practicing relaxation techniques, introducing previously unfamiliar relaxation techniques and mindfulness practices
- Summary of relaxation techniques
  Principles of using relaxation techniques
  Awareness triangle and positive self-talk
- Handling disclosure issues
  Relevant laws, principles of disclosure, importance of social support networks
  Specific considerations when disclosing to different people (medical staff, friends, family, and partners)
  Tips for disclosure
- Relaxation techniques
  Awareness of body scan

**Week 6 (web-based)**

- How to protect those around you
  Common questions and misconceptions
  Explaining HIV transmission using examples from daily life
- Relaxation in dynamic situations
  Importance of emotional management and awareness of dynamic and sensory awareness
- Relaxation techniques
  Dynamic awareness of neck stretching

**Week 7 (web-based)**

- Work-related issues
  Conveying the belief in the ability to work, and related experiences of previous people living with HIV/AIDS
  How to obtain a health certificate and which physical examinations will test for HIV
- Obtaining and handling information
  Principles of handling information and examples of sources of correct information
- Relaxation techniques
  Sensory awareness

**Week 8 (web-based)**

- How to control emotions
  Importance of emotional control and small techniques
- Future planning
  Adjusting mindset for the future
  Answers about stable relationships and the possibility of having children
- Relaxation techniques
  Mountain meditation

**Week 9 (web-based)**

- Review summary
  Summarizing and answering previous web-based consultation questions from people living with HIV/AIDS
- Summary of previous interventions
  Review the content of all intervention programs
- Relaxation techniques
  Gratitude meditation

**Week 10 (offline)**

- Summary
  Understanding the situation of people living with HIV/AIDS in the past 2 months
  Review the content of all intervention programs

#### The First Individual Session

The first individual session aimed to establish a robust relationship with participants through personalized conversations focused on the adaptation tasks identified in the baseline survey. Conducted in a private and tranquil office setting immediately following allocation, this session lasted from 30 to 60 minutes. The session consisted of the following components:

- An individualized PowerPoint (Microsoft Corp) presentation and dialogue on the AiCare program, life expectancy, and the significance of ART adherence.
- Instruction on using a task-based checklist to address HIV-related issues in daily life.
- Practice of a relaxation technique selected from a menu of options, under the interventionist’s guidance.
- An open communication segment for participants to seek additional counseling as desired.

#### Web-Based Sessions

All the web-based sessions were delivered through the interventionist’s personal and official WeChat account. Each Monday, two illustrated essays were shared via the official account, with themes curated from our preliminary interviews with people living with HIV/AIDS [41]. Accompanying the essays was an audio component, consisting of one or two short recordings (6-13 minutes in length), created by the interventionist to instruct participants in relaxation techniques.

Counseling was provided through individual WeChat messages. Additionally, the interventionist contacted participants every Saturday to assess their engagement with the web-based sessions. This was done by asking two simple questions about the essays to gauge how often and how well the participants understood the content.

Furthermore, a WeChat group was established for voluntary participation. This group provided a platform for weekly discussions related to the essays and an open forum for participants to discuss topics of their choosing, with an emphasis on adhering to principles of privacy and mutual respect.

#### The Second and Third Individual Sessions

The second individual session was conducted in the fifth week, either in person during participants’ clinic visits or via WeChat video or voice calls. The third individual session took place in the tenth week, conducted in person. In the second individual session, the interventionist guided participants in practicing relaxation techniques and engaged in dialogue regarding their adaptive tasks over the preceding month. Additionally, the third individual session involved a comprehensive dialogue with participants about their experiences over the past 9 weeks, addressing any questions and providing guidance on how to integrate the knowledge and techniques learned into their future daily lives.

### Outcomes

We adopted a battery of questionnaires that assessed QoL, acceptance of illness, mental health outcomes (anxiety and depression), and ART adherence at T0, T1, and T2. Besides, a self-designed questionnaire was used to evaluate the feasibility of the AiCare program.

### Primary Outcome

The primary outcome measure was QoL, assessed using the World Health Organization Quality of Life Instrument for HIV Infection [42]. This instrument consists of 31 items, with 2 items dedicated to evaluating overall QoL and health. The remaining 29 items are categorized across 6 domains: physical health, psychological health, independence, social relationships, environmental health, and personal beliefs. Each item is scored on a 5-point Likert scale, and a composite score is derived following the prescribed guidelines [42], with higher scores indicating superior QoL.

### Secondary Outcomes

#### Overview

Secondary outcomes included changes in three pivotal areas: acceptance of illness, assessed by the AIS; symptoms of anxiety and depression, assessed by the PHQ-4; and ART adherence, assessed by the CASE Index.

#### AIS

Developed by Felton et al [43], the AIS is a well-established instrument that comprises eight items evaluating the extent to which participants accept HIV without negative experiences. Responses are scored on a 5-point Likert scale, where 1 indicates strong agreement and 5 indicates strong disagreement. A higher total score reflects greater acceptance of illness, with scores below 30 indicating suboptimal acceptance.

#### PHQ-4

The PHQ-4 is a succinct yet robust tool for assessing anxiety and depression symptoms [44]. It includes four items, with the first two targeting anxiety and the latter two targeting depression. The scale inquires about the frequency of experiences related to each item, using a 4-point Likert scale that ranges from 0=not at all to 4=nearly every day. A total score of 3 or higher in either domain or on the total scale indicates the presence of anxiety, or depression, or suboptimal mental health.

#### The CASE Index

The CASE Index is a validated instrument for monitoring ART adherence, using a composite score derived from three straightforward, self-reported questions regarding ART adherence: the difficulty in taking ART medication on schedule, the average number of days per week with at least one missed dose, and the most recent occasion on which the patient missed at least one dose. The score ranges from 3 to 16, with higher scores signifying better adherence [45]. Patients attaining a CASE Index Score of >10 were defined as having optimal adherence.

### Statistical Analysis

Statistical analysis was performed with the SPSS (version 25.0), following an intention-to-treat approach. Descriptive statistics were calculated as means and SDs for continuous variables and as frequencies and percentages for categorical variables. Baseline characteristics of the two groups were compared using independent *t* tests for normally distributed continuous variables, Mann-Whitney *U* tests for nonnormally distributed continuous variables, and chi-square tests for categorical variables. The Shapiro-Wilk test was used to assess normality.

To evaluate the intervention effects, we used generalized estimating equations (GEE) with a linear scale response and difference-in-difference (DID) analysis. A two-sided *P* value of less than .05 was considered statistically significant. The GEE models included fixed effects for time, group, and their interaction, using an exchangeable correlation structure to account for within-subject correlations. The DID estimates were calculated as the change in values before and after baseline (eg, QoL _postintervention_–QoL_preintervention_) in the intervention group minus the change in values from baseline in the control group adjusting for baseline characteristics [46,47]. The size and direction of a DID estimate indicate the magnitude and direction of the change in the outcome for the intervention group, accounting for changes observed in the control group.

### Ethical Considerations

This study was approved by the Institutional Review Board of Xiangya Nursing School, Central South University (2018034). All participants provided a written informed consent form. Those invited to participate in the study were informed of the content, purpose, and benefits of participating. Patients were aware of their rights to withdraw or discontinue participation in the study at any time. A compensation of RMB 50 Yuan (US $6.91) was given after each assessment for each participant. The collected information was kept confidential and only used for research purposes.

## Results

### Study Participants

**Figure 1:**
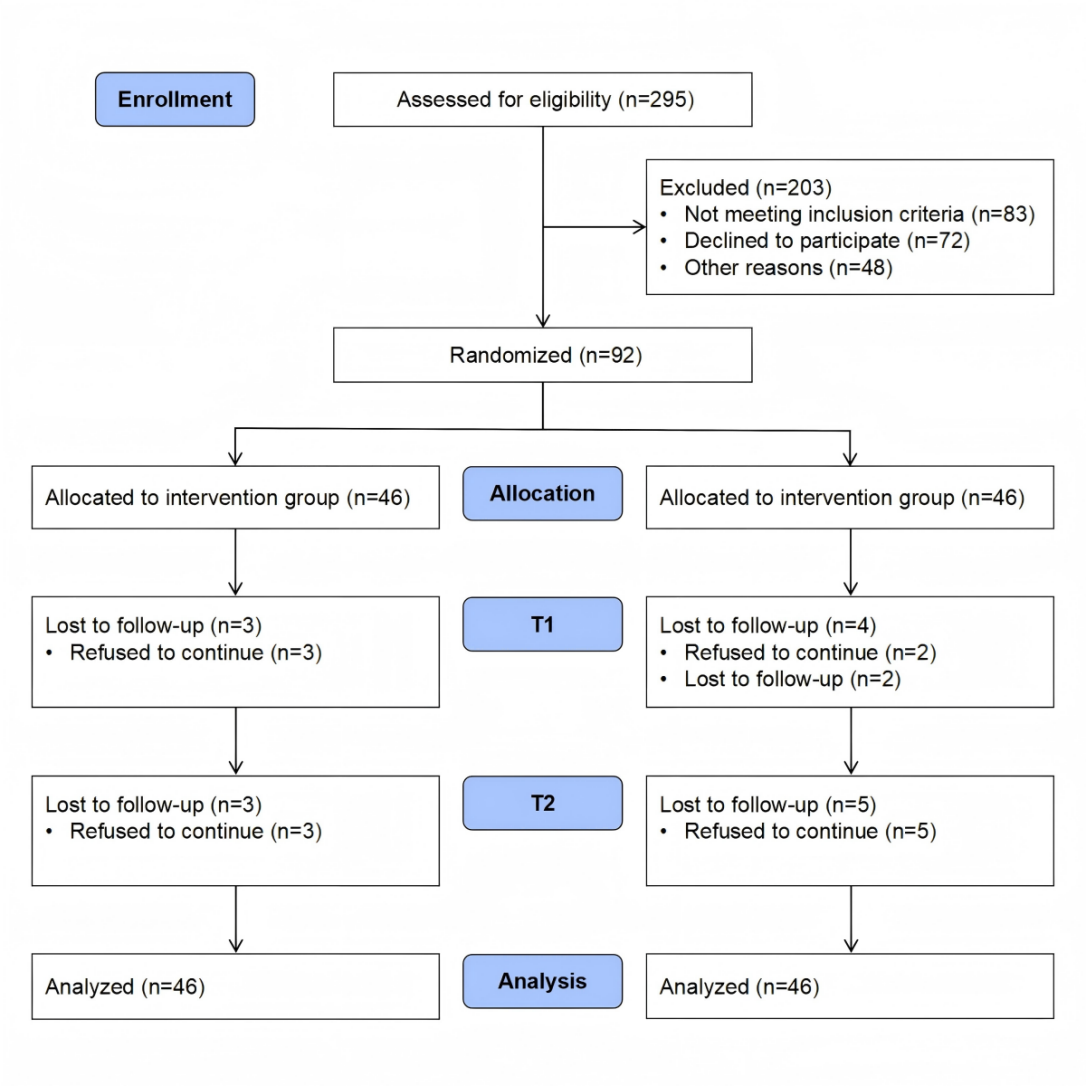
CONSORT (Consolidated Standards of Reporting Trials) diagram of the trial. T1: post intervention; T2: 3 months post intervention.

We evaluated 295 individuals for eligibility, excluding 83 individuals who did not meet the inclusion criteria and 120 individuals who declined to participate. This resulted in 92 participants being randomly assigned to either the intervention group (n=46) or the control group (n=46). During follow-up, 15 participants (6 from the intervention group and 9 from the control group) were lost to follow-up, and they were comparable to the remaining 77 participants. Details are shown in Figure 1.

The baseline characteristics of the study participants are presented in Table 1. No significant differences were observed between the groups, except for age and duration of ART. Therefore, we included age and duration of ART treatment as covariates in the subsequent analysis of the study effects. The median age of participants in the intervention group was 29 (IQR 22-35) years, with an age range spanning from 18 to 62 years, in contrast to the control group, which had a median age of 32 (IQR 26-40) years, with an age range from 18 to 60 years. Furthermore, the intervention group had a median duration of ART treatment of 17 (IQR 5-34) months, whereas the control group demonstrated a significantly longer median duration of 36 (IQR 16-60) months.

**Table 1:** Baseline characteristics of participants in the intervention and control groups.

		Intervention group (n=46)	Control group (n=46)	Chi-square (*df*)/*Z*^a^	*P* value
Age (years), median (IQR)		29 (22-35)	32 (26-40)	2.35	.02
Ethnicity (Han/Other), n (%)		43 (94)	46 (100)	N/A^b^	.24
Male, n (%)		39 (85)	37 (80)	.30 (1)	.58
Live in the countryside, n (%)		27 (59)	26 (57)	.05 (1)	.83
**Educational level, n (%)**				2.72 (4)	.61
	Below junior high school	2 (4)	2 (4)		
	Junior high	5 (11)	7 (15)		
	High school	6 (13)	10 (22)		
	College or secondary school	16 (35)	10 (22)		
	Bachelor’s degree or above	17 (37)	17 (37)		
No religious beliefs, n (%)		39 (85)	40 (87)	.09 (1)	.77
**Marital status, n (%)**				1.26 (4)	.60
	Married	16 (35)	21 (46)		
	Divorced or widowed or spouse or separation	4 (9)	4 (9)		
	Single	26 (57)	21 (46)		
Live alone, n (%)		14 (30)	16 (35)	.20 (1)	.66
Employed, n (%)		34 (74)	35 (76)	N/A	.81
**Sexual orientation, n (%)**				N/A	.86
	Heterosexuality	11 (24)	12 (26)		
	Homosexual	29 (63)	26 (57)		
	Bisexual	4 (9)	4 (9)		
	Uncertain	2 (4)	4 (9)		
**Personal monthly income** **(Yuan; 1 Yuan=US $0.14), n (%)**				N/A	.43
	No income	12 (26)	10 (11)		
	≤1000	2 (4)	2 (4)		
	1001-2000	3 (7)	0 (0)		
	2001-3000	5 (11)	3 (7)		
	3001-4000	3 (7)	4 (9)		
	>4000	16 (35)	19 (41)		
	Instability	3 (7)	8 (17)		
**Family monthly income (Yuan), n (%)**				N/A	.33
	0-5000	7 (15)	11 (24)		
	5001-10,000	9 (20)	14 (30)		
	10,001-15,000	12 (26)	9 (20)		
	15,001-20,000	2 (4)	4 (9)		
	20,001-25,000	4 (9)	2 (4)		
	25,001-30,000	3 (7)	0 (0)		
	>30,000	9 (20)	6 (13)		
Duration of HIV infection, median (IQR)		22 (6-43)	36 (16-60)	1.58	.11
Duration of ART^c^, median (IQR)		17 (5-34)	29 (14-44)	N/A	.02
Side effects of ART, n (%)		18 (39)	13 (28)	1.22 (1)	.38
HIV disclosure to others		7 (15.2)	7 (15.2)	0.00 (1)	≥.99

^a^The statistical tests used for the comparisons include the chi-square test, Fisher exact test, or Mann-Whitney *U* test, depending on the nature of the data and the assumptions met for each analysis.

^b^Not applicable.

^c^ART: antiretroviral therapy.

### Effect of AiCare Program on Primary Outcome—QoL

Regarding the changes of QoL, the GEE analysis showed the interaction of time and intervention was significant (*χ*^2^_5_=11.4; *P*=.003), while the main effect of time (*χ*^2^_2_=1.4; *P*=.50) and intervention (*χ*^2^_1_=0.1; *P*=.71) were not. As in Table 2, subsequent analyses indicated a notable improvement in QoL for the hybrid intervention group following the intervention.

The DID model in Table 2 showed that, at T1, QoL scores in the intervention group increased by 6.35 compared to the control group (95% CI 2.62-10.93; *P*=.001). However, no statistically significant difference in QoL scores was observed at T2.

**Table 2:** The QoL^a^ score and intervention effect of the two groups.

	Intervention group (n=46), mean (SD)	Control group (n=46), mean (SD)	DID^b^	
			β (95% CI)	*P* value
T0^c^	78.9 (11.7)^d^	81.2 (12.5)	N/A^e^	N/A
T1^f^	82.9 (13.2)^d^	78.8 (14.2)	6.35 (2.62 to 10.93)	.001
T2^g^	80.4 (14.6)	79.1 (15.0)	3.62 (–1.53 to 8.77)	.17

^a^QoL: quality of life.

^b^DID: difference-in-difference.

^c^T0: baseline.

^d^Indicates significant differences in scores marked with the same footnotes.

^e^Not applicable.

^f^T1: post intervention.

^g^T2: 3-month follow up.

### Effect of AiCare on Secondary Outcomes

#### Acceptance of Illness

In our study examining acceptance of illness, the GEE analysis revealed a significant interaction effect between time and intervention on acceptance of illness scores (*χ*²_5_=–16.1; *P*<.001). Although the main effects of time (*χ*²_2_=–4.1; *P*=.13) and intervention (*χ*²_1_=–3.1; *P*=.08) alone were not significant, the hybrid intervention group showed a substantial improvement in acceptance scores, as demonstrated in Table 3.

The DID model in Table 3 showed that disease acceptance increased by 4.49 in the intervention group, compared to the control group at T1 (95% CI 2.29-6.68; *P<*.001). At T2, disease acceptance increased by 3.65 in the intervention group compared to the control group (95% CI 1.22-6.08, *P*=.003).

**Table 3:** The acceptance of illness score and intervention effect of the two groups.

	Intervention group (n=46), mean (SD)	Control group (n=46), mean (SD)	DID^a^	
			β (95% CI)	*P* value
T0^b^	24.9 (5.31)^cd^	25.5 (5.02)	N/A^e^	N/A
T1^f^	30.0 (6.13)^c,g^	24.1 (6.38)^g^	4.49 (2.29-6.68)	<.001
T2^h^	28.0 (6.75)^d,i^	25.0 (6.63)^i^	3.65 (1.22-6.08)	.003

^a^DID: difference-in-difference.

^b^T0: baseline.

^c,d^Indicates significant differences in scores marked with the same footnotes.

^e^Not applicable.

^f^T1: post intervention.

^g^Indicates significant differences in scores marked with the same footnotes

^h^T2: 3-month follow up.

^i^Indicates significant differences in scores marked with the same footnotes.

#### Mental Health (Anxiety and Depression)

The comparative analysis of PHQ-4 scores between the hybrid intervention and control groups revealed a significant interaction effect of time and intervention (*χ*²_5_=–19.4; *P*<.001), whereas the main effects of time (*χ*²_2_=–4.3; *P*=.11) and intervention (*χ*²_1_=–0.6; *P*=.46) were not significant. This indicates that the trends in psychological health levels differed between the two groups over time.

The DID model in Table 4 shows that, at T1, the incidence of anxiety symptoms in the intervention group decreased by 1.98 compared to the control group (95% CI 0.63-3.34; *P*=.004). Although the incidence of depression symptoms in the intervention group also decreased, the difference was not statistically significant. At T2, the incidence of anxiety symptoms in the intervention group decreased by 2.054 compared to the control group (95% CI 0.57-3.54; *P*=.007). Similarly, while the incidence of depression symptoms in the intervention group decreased, the change was not statistically significant.

**Table 4:** Comparative analysis of anxiety and depression.

		Intervention group (n=46), n (%)	Control group (n=46), n (%)	DID^a^	
				β (95% CI)	*P* value
**Anxiety**					
	T0^b^	15 (33)^c,d,e^	7 (15)^e^	N/A^f^	N/A
	T1^g^	5 (12)^d^	11 (26)	1.98 (0.63 to 3.34)	.004
	T2^h^	4 (10)^c^	9 (24)	2.05 (0.57 to 3.54)	.007
**Depression**					
	T0	14 (30)	8 (17)	N/A	N/A
	T1	8 (19)	10 (24)	1.04 (–0.10 to 2.19)	.07
	T2	7 (18)	9 (24)	1.15 (–0.16 to 2.45)	.09

^a^DID: difference-in-difference.

^b^T0: baseline.

^c,d,e^Indicate significant differences in scores marked with the same footnote.

^f^Not applicable.

^g^T1: post intervention.

^h^T2: 3 months post intervention.

#### ART Adherence

The analysis revealed no significant interaction effect between time and intervention (*χ*²_5_=3.5; *P*=.17), nor were there significant main effects attributable to time (*χ*²_2_=2.1; *P*=.35), or intervention alone (*χ*²_1_=0.3; *P*=.61). This indicates the impact of the intervention on ART adherence was not statistically significant.

### Feedback Toward the AiCare Program

Among the 46 participants enrolled in the intervention group, 94% (n=43) adhered to the AiCare program. A detailed examination of the offline components reveals that all 46 participants successfully completed the first session. Subsequently, 31 participants completed the second individual-to-individual session during their visit to the HIV clinic, and 12 participants finished via video call. For the third offline session, 29 participants completed it within the clinic setting, and 14 participants did so remotely via video call. The average number of offline interactions was 2.86 (SD 0.35). Regarding the web-based modules, among the 43 participants, 16 (37%) individuals successfully completed all the modules. Participation spanned a range of 5 to 8 modules, with a median number of completed modules being 7 (IQR 7-8).

Table 5 provides a detailed breakdown of how web-based and offline modules were assessed, with the data indicating an overall favorable evaluation of the AiCare program. In the overall evaluation, the majority of participants found the program’s arrangement to be reasonable and considered it worthy of promotion.

**Table 5:** Evaluation of the AiCare^a^ program.

Evaluation			Actual score range		Mean (SD)
**Web-based module**					
	The information web-based is useful	4~5		4.60 (0.50)	
	The web-based information is what I need	4~5		4.51 (0.51)	
	The information web-based is very comprehensive	3~5		4.37 (0.69)	
	Web-based tips are useful	3~5		4.33 (0.68)	
	Web-based tips are what I need	3~5		4.14 (0.77)	
	The web-based modules are what I like	3~5		4.37 (0.62)	
**Offline module**					
	Meeting and talking offline can help me solve some problems	3~5		4.44 (0.59)	
	The information provided by the offline meeting is what I need	3~5		4.47 (0.55)	
	The offline meeting provides the skills is what I need	3~5		4.23 (0.72)	
	The way I meet and talk offline is I like	3~5		4.33 (0.68)	
**Overall plan**					
	Overall, the arrangement of the project is sound	3~5		4.33 (0.68)	
	Overall, the arrangement of the project is worth popularizing	3~5		4.56 (0.55)	

^a^AiCare: Adaptation intervention with Comprehensive-task disease management framework to achieve renormal life.

## Discussion

### Principal Findings

We explored a comprehensive task-based hybrid intervention called AiCare to promote the adaptation outcomes of people living with HIV/AIDS. The results showed AiCare program significantly facilitated people living with HIV/AIDS in attaining a more harmonious state of coexistence with HIV, evidenced by enhanced QoL, greater acceptance of illness, and improved mental health. Furthermore, participant feedback suggests that the AiCare program is well-designed and effectively assists people living with HIV/AIDS in improving their disease adaptation outcomes, warranting its potential feasibility [16].

The AiCare program has demonstrated significant potential to improve comprehensive outcomes for this population, particularly in terms of QoL and acceptance of illness. The intervention covers five critical aspects, namely physical, psychological, social, spiritual, and occupational, which collectively enhance participants’ coping mechanisms and overall QoL. This holistic approach aligns with the core hypothesis of the comprehensive task-based model, which posits a direct relationship between disease adaptation and multifaceted task completion in people living with HIV/AIDS [16]. Similar to a study on patients’ adaptation to cancer [48], the AiCare program highlighted the importance of psychological tasks during the adaptation process, and provides extensive information on relaxation techniques and psychological adjustment, enhancing patients’ psychological flexibility. This means that even when people living with HIV/AIDS encounter HIV-related problems, these issues in the five aspects have less impact on their lives [49], further improving their acceptance of the disease and facilitating QoL [50].

The findings of this study underscore the promising feasibility of the AiCare program. Our participants, ranging in age from 18 to 62 years, expressed a profound willingness to recommend the program to their peers. Despite a minor attrition rate, there were no significant differences in the characteristics of those who completed the program versus those who did not, highlighting the potential suitability of the AiCare program for older people living with HIV/AIDS, especially those older than 50 years. One likely reason for this success is the initial offline module, which fostered a sense of trust and familiarity between participants and the interventionist. This foundational interaction facilitated a smooth transition to, and greater acceptance of, the subsequent web-based modules. Additionally, the second individual-to-individual module played a critical role in maintaining a high retention rate by addressing challenges participants faced during the web-based modules, further consolidating the trust relationship. This is supported by evidence from older patients with chronic obstructive pulmonary disease [51] and studies conducted in internet hospitals [52], where offline experiences have been shown to enhance web-based engagement and service quality.

Though the long-term effect on acceptance of illness is significant, the AiCare program had limited long-term effects on improving QoL, potentially due to the insufficient frequency of both web-based and offline modules. For instance, a previous intervention for this population was conducted web-based 3 to 5 times per week [15], whereas the web-based component in this study was only twice per week. According to the positive self-management framework, the reduced frequency might have hindered improvements in self-efficacy. As a result, when participants encountered new disease-related tasks post intervention, the necessary behavioral and cognitive coping strategies decreased. Additionally, people living with HIV/AIDS encounter common tasks, but the nature and severity of these tasks can vary significantly among individuals [53]. The AiCare program, which included only three individualized modules, may not have provided enough support to address each participant’s specific proximal tasks. This limitation could impact the effectiveness observed 3-month follow-up.

This study demonstrated the feasibility and effectiveness of a comprehensive task-based intervention in enhancing adaptation outcomes among people living with HIV/AIDS. The AiCare program shows promise for implementation in clinical settings, particularly with the use of hybrid modules. However, further research is needed to determine how to incorporate more individualized modules cost-effectively while ensuring that it has a long-term impact. Given the potential of artificial intelligence in providing personalized interventions, exploring its feasibility for use among people living with HIV/AIDS in future studies could be valuable.

### Limitations

Despite the strengths of this study, several limitations must be acknowledged. First, the study was conducted at a single center with a small sample size who experienced difficulties in the adaptation process, and 84.8% (n=92) of the participants were male, which limits the generalizability of the findings. Especially, the potential feasibility of the AiCare program for old people living with HIV/AIDS should be further explored. Second, the study involved eight web-based modules, which theoretically posed a risk of information leakage among participants, potentially contaminating the study’s outcomes. To mitigate this risk, participants were explicitly instructed not to share module information prior to the intervention. Given that the intervention was multifaceted, including both offline and web-based modules, any contamination, if it occurred, is likely to have had a minimal impact on the overall efficacy of the AiCare program. Additionally, though we adopted anonymous surveys and encouraged honest and uninhibited feedback from participants, the results may have been influenced by ceiling effects and social desirability bias, which could limit the ability to detect subtle differences in participants’ experiences and compromise the reliability of the findings.

### Conclusions

The AiCare program, a comprehensive task-based hybrid intervention, has illustrated its capacity to significantly bolster disease adaptation outcomes among people living with HIV/AIDS, notably in enhancing QoL, acceptance of illness, and mental health, and particularly in reducing anxiety symptoms. Given the limitations of the study, future research should investigate a hybrid intervention that incorporates more individualized modules and a higher dose of both web-based and offline components, aiming to secure enduring benefits in the disease adaptation process for a more diverse cohort of people living with HIV/AIDS.

## Appendix

Multimedia Appendix 1 CONSORT-eHEALTH checklist (V 1.6.1).

## Abbreviations

AiCare: Adaptation intervention with Comprehensive-task disease management framework to achieve renormal life
AIS: Acceptance of Illness Scale
ART: antiretroviral therapy
CASE: Center for Adherence Support Evaluation
CONSORT: Consolidated Standards of Reporting Trials
DID: difference-in-difference
GEE: generalized estimating equation
mHealth: mobile health
PHQ-4: Patient Health Questionnaire-4
QoL: quality of life
T0: baseline
T1: post intervention
T2: 3 months post intervention

## Data Availability

The datasets generated during and analyzed during this study are available from the corresponding author upon reasonable request.
